# Unraveling the ROS-Inflammation-Immune Balance: A New Perspective on Aging and Disease

**DOI:** 10.14336/AD.2024.1253

**Published:** 2026-01-03

**Authors:** Sihang Fang, Mingjun Jiang, Juan Jiao, Hongye Zhao, Dizhi Liu, Danni Gao, Tenger Wang, Ze Yang, Huiping Yuan

**Affiliations:** ^1^The Key Laboratory of Geriatrics, Beijing Institute of Geriatrics, Institute of Geriatric Medicine, Chinese Academy of Medical Sciences, Beijing Hospital/National Center of Gerontology of National Health Commission, Beijing, China.; ^2^Respiratory Department, Beijing Children’s Hospital, Capital Medical University, China National Clinical Research Center of Respiratory Diseases, National Center for Children’s Health, Beijing, China.; ^3^Department of Clinical Laboratory, the Seventh Medical Center of PLA General Hospital, Beijing, China.; ^4^Department of Biochemistry and Molecular Biology, The Key Laboratory of Neural and Vascular Biology, Ministry of Education of China, Hebei Medical University, Shijiazhuang, Hebei, China.

**Keywords:** entropy, oxidative stress, inflammaging, immunosenescence, age-related disease, antiaging interventions

## Abstract

Increased entropy is a common cause of disease and aging. Lifespan entropy is the overall increase in disorder caused by a person over their lifetime. Aging leads to the excessive production of reactive oxygen species (ROS), which damage the antioxidant system and disrupt redox balance. Organ aging causes chronic inflammation, disrupting the balance of proinflammatory and anti-inflammatory factors. Inflammaging, which is a chronic low-grade inflammatory state, is activated by oxidative stress and can lead to immune system senescence. During this process, entropy increases significantly as the body transitions from a state of low order to high disorder. However, the connection among inflammation, aging, and immune system activity is still not fully understood. This review introduces the idea of the ROS-inflammation-immune balance for the first time and suggests that this balance may be connected to aging and the development of age-related diseases. We also explored how the balance of these three factors controls and affects age-related diseases. Moreover, imbalance in the relationship described above disrupts the regular structures of cells and alters their functions, leading to cellular damage and the emergence of a disorganized state marked by increased entropy. Maintaining a low entropy state is crucial for preventing and reversing aging processes. Consequently, we examined the current preclinical evidence for antiaging medications that target this balance. Ultimately, comprehending the intricate relationships between these three factors and the risk of age-related diseases in organisms will aid in the development of clinical interventions that promote long-term health.

## 1. Introduction

As human life expectancy has risen in recent decades, the worldwide aging population has expanded, bringing about greater incidence of age-related dysfunction and diseases. According to The Lancet Public Health, 51.3% of the global disease burden is due to age-related illnesses [1]; therefore, identifying the underlying mechanisms of aging, decreasing morbidity and mortality in elderly individuals, and developing healthy aging strategies are necessary. In his book "The Mysteries of Biology," the Nobel Prize-winning biologist Peter Medawar proposed two types of aging in the body: inherent aging, which is aging as a biological necessity, and "wear and tear" aging, which is caused by the "cumulative effect of cyclic stress". The former type of aging is biological in nature, whereas the latter type of aging is based in physics. The body functions as an open system characterized by a highly organized dissipative structure that exchanges matter and energy with the outside world to achieve a negative entropic flow, thereby offsetting the body’s entropy increase and transforming the body from a chaotic and disordered state to a stable ordered state [2].

The process of aging is marked by a continuous deterioration of physiological integrity, causing functional impairment and greater susceptibility to mortality. This decline is the main risk factor for typical age-related diseases, including cardiovascular disorders, diabetes, cancer, neurodegenerative diseases, and autoimmune diseases. In recent years, accumulating evidence has shown a strong association among aging-associated pathologies, the tissue inflammatory environment, and immune functional disorders. Clinical evidence has established that chronic low-grade inflammation, which is also known as inflammaging, is among the leading causes of age-related morbidity and mortality [3]. Among the various molecular alterations linked to aging, the production of reactive oxygen species (ROS) has emerged as a feature of aging across multiple species, indicating its vital role. Moreover, the impact of age on immunity is unclear, and many dysregulated responses can be attributed to immunosenescence, which is based on the impairment of stem cells [4]. These three mechanisms involve intricate collaborative connections and affect the onset and progression of aging. The importance and interconnection of these mechanisms are evident, yet their role in regulating aging and contributing to age-related diseases is still not fully understood. Therefore, further investigations and discussions are needed.

**Figure 1.**
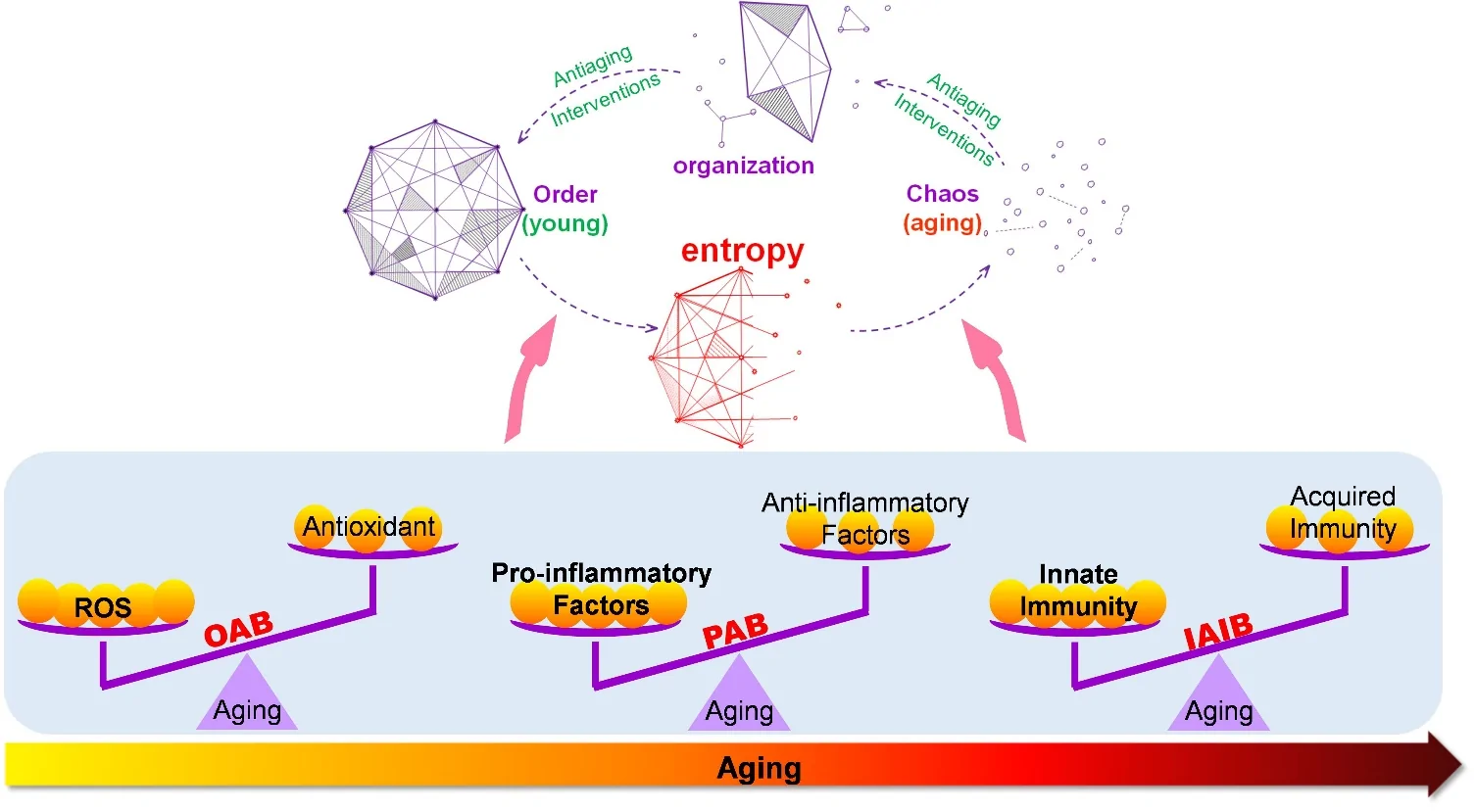
**Relationship between increased entropy and the ROS-inflammation-immune balance in the process of aging**. Aging leads to the excessive production of ROS, which damages the antioxidant system and disrupts redox balance, resulting in the occurrence of oxidative stress. Organ aging causes chronic inflammation, disrupting the balance of proinflammatory and anti-inflammatory factors and leading to inflammaging. With the accumulation of oxidative and inflammatory stress, innate immunity outweighs adaptive immunity, leading to the disruption of immune balance and causing immunosenescence. The ROS-inflammation-immune balance, which consists of oxidative stress, inflammation and immunosenescence, is an important common mechanism in the aging process. Moreover, imbalance in the relationship described above disrupts the regular structures of cells and alters their functions, leading to cellular damage and the emergence of a disorganized state marked by increased entropy. Lifespan entropy is the overall increase in disorder caused by a person over their lifetime. Higher order is indicative of lower entropy and better health/youth, whereas lower order is indicative of higher entropy, worse health/aging, and potentially more serious diseases. Aging and illness can increase body entropy, affecting organ entropy and overall health. Increased entropy is a common factor in human disease and aging, making it important to use antiaging interventions to maintain a low-entropy state and prevent or reverse pathological processes.

Entropy theory, which is rooted in thermodynamics, describes the tendency of physical and chemical systems to progress from states of order to states of disorder. This principle is applicable in not only physics but also biological systems. Lifetime entropy is the amount of entropy an individual generates during their lifetime. Health is a low-entropy, highly ordered state that is maintained through complex regulatory systems, such as redox balance, inflammation control, and immune regulation, that preserve homeostasis. Conversely, in a disease state, the internal structure and function of the body are damaged and can enter or be in a chaotic and disordered high-entropy state (Fig. 1). The greater the degree of order of a system, the lower its entropy value, and the better its health. Conversely, the more disorder there is in the body, the greater the entropy value of the system, and the worse the health status. As people age or become ill, their body entropy increases, leading to higher entropy both overall and in specific organs [5]. In mammals, a decline in biological functions due to increasing entropy at the epigenetic level has been observed in processes such as the breakdown of DNA methylation patterns and histone modifications, leading to a loss of cellular identity and contributing to the aging process. Entropy results in aging-related physiological declines, including inflammation and immune dysregulation [6]. Entropy theory plays a fundamental role in explaining the process of aging and the degradation of biological systems, as it is directly related to information loss and the transition from a nonequilibrium state to an equilibrium state [7, 8]. Diseases such as metabolic disorders, metabolic syndrome, and metabolic inflammation have shown an increase in entropy.

The body absorbs elements from the external environment and generates negative entropy to offset the internal increase in entropy. This process involves the ingestion of nutrients and pathogenic factors, leading to the production of harmful substances and errors in DNA replication, gene transcription, and biochemical synthesis while maintaining the body's structure and function. The body maintains a state of low entropy via self-defense mechanisms such as antioxidants, inflammation, the immune response, the stress response, autophagy, and apoptosis.

In this review, we extend the concept of inflammaging to a theory of ROS-inflammation-immune balance and examine aging from the perspective of the entropy increase generated during this process. By examining the relationships among oxidative stress, inflammaging and immunosenescence, we propose novel links among these three processes. We describe molecular mechanisms that associate oxidation, inflammation and immunosenescence and illustrate how these processes regulate tissue health and homeostasis, as well as their potential implications for antiaging therapeutic interventions. We believe that the intricate connections between inflammaging-associated processes and the resulting increase in entropy are important for understanding the mechanisms of aging.

## 2. Redox Balance

Mitochondria often produce free radicals during metabolism. The antioxidant system maintains a state of redox balance. Oxygen (O_2_) is essential for all aerobic organisms because it plays a vital role in the synthesis of adenosine triphosphate (ATP), which supports life-maintaining metabolic processes. In general, when an organism is in redox equilibrium, oxidants and antioxidants are in dynamic balance, maintaining internal homeostasis. However, oxygen can also cause damage by producing ROS when oxygen reduction is insufficient. An excess of ROS can overwhelm the antioxidant system, leading to a disruption of redox balance. The increased levels of free radicals can disrupt normal cellular structures and alter cellular functions, ultimately resulting in cellular impairment and the emergence of a chaotic state characterized by increased entropy [5]. Although the body undergoes repair via pathways such as apoptosis and autophagy, cellular damage gradually accumulates, causing damage to cellular defense mechanisms and apoptotic mechanisms. Senility may be driven by the heightened reactivity of free radicals and ROS generated in cells with increasing age, which consequently leads to deleterious oxidation and cellular damage [9]. Additionally, natural aging processes in organs can result in inflammation. Research shows that fragmented mitochondria, often observed in aging cells, are associated with impaired bioenergetic capacity and increased ROS production, both of which contribute to cellular entropy [10]. Mitochondrial damage can diminish cellular energy production and hinder DNA repair, creating a detrimental cycle that increases the risk of age-related health issues. Excessive production of ROS leads to oxidative damage to macromolecules, such as lipids, proteins, and DNA, disrupting cellular structures and altering physiological functions. This chemical modification induces physical changes, including membrane destabilization and protein misfolding, which contribute to a transition from a low-entropy, organized state to a high-entropy, disordered state [11]. The Gompertz-Makeham model was developed to corroborate the trajectory of the human lifespan.

## 2.1. Oxidants and Antioxidants: Two Sides of Redox Balance

The redox balance in organisms depends on maintaining the dynamic balance between ROS generation and elimination, which requires intricate oxidative and antioxidative systems.

ROS mainly includes hydroxyl radical (OH^-^), superoxide anion (O_2_^-^), hydrogen peroxide (H_2_O_2_), and peroxynitrite (ONOO^-^). O_2_^-^ is formed when oxygen captures one electron and generates H_2_O_2_ and OH^-^ [12]. In normal cells, mitochondria and NADPH oxidases (NOXs) are two major sources of ROS production. Mitochondria generate ROS as a byproduct of oxidative phosphorylation, primarily within the electron transport chain. In addition, NOXs produce ROS in the membranes of cell compartments, including the endoplasmic reticulum (ER), nucleus, and peroxisomes [8], which contribute significantly to the level of cellular ROS in eukaryotic cells [13]. In innate immune cells like macrophages, NOX2, part of the NOX family, is essential for generating extracellular ROS, vital for immune defense [13]. Additional mechanisms that may play a role in ROS formation include immune activation, xanthine oxidase (XO), and arachidonic acid (AA) metabolism [14, 15].

Although ROS has beneficial effects, they need to be neutralized immediately to avoid excessive ROS accumulation, which can result in oxidative damage in cellular components. A variety of antioxidant processes have evolved to either inhibit the creation of ROS or to neutralize them once they are formed. Enzymatic antioxidants include superoxide dismutase (SOD), which converts O_2_^-^ into H_2_O_2_, and glutathione peroxidase (GPX) and catalase, which catalyze the decomposition of H_2_O_2_ into H_2_O and O_2_ [15]. Additionally, non-enzymatic antioxidants, such as vitamins A, C, and E; melatonin; and polyphenols, are essential in regulating ROS levels and maintaining oxidative balance [16]. Overall, the equilibrium between oxidant and antioxidant compounds helps keep ROS at proper levels and sustains homeostasis.

## 2.2. Oxidative Stress: The Initiator of Aging

A temporary rise in ROS production can trigger an adaptive response by upregulating antioxidant expression [17-19]; thus, oxidative stress can be quickly resolved. As mitochondrial function decreases with age, electron leakage from the respiratory chain may occur, resulting in the generation of superoxide radicals. Fragmented mitochondria are less capable of maintaining membrane potential, contributing to a cascade of dysfunction that includes increased ROS production and decreased ATP generation, driving cellular entropy [13]. Moreover, the high activity of SOD and monoamine oxidase B results in the greater production of H_2_O_2_ [20]. Thus, through the uncontrolled production of oxidants and/or impaired antioxidant defenses, the level of ROS exceeds the available antioxidant protection, which disrupts the balance and leads to oxidative stress. In vivo, mice with impaired immune function and excessive oxidative stress exhibit an inadequate response to stress, similar to that of old mice, and have a shorter lifespan [21, 22]. A 20% extension in lifespan was seen in vitro with the upregulation of mitochondrial antioxidant and catalase expression [23]. The ER is central to maintaining protein homeostasis, and disruptions in ER function lead to the initiation of the unfolded protein response (UPR). Chronic ER stress, commonly observed in aging and age-related diseases, leads to sustained UPR activation, resulting in maladaptive cellular outcomes such as apoptosis and inflammation. This chronic stress state is associated with increased cellular entropy, as the misfolding and accumulation of proteins create a more disordered and less efficient cellular environment [10].

## 2.3. Genome Damage Caused by ROS

Chronic ROS accumulation causes long-term oxidative stress, creating a pro-oxidant environment, subsequently causing DNA damage, functional loss, and cell death, ultimately accelerating aging. Among the sources of ROS production, generation of mitochondrial ROS (mitROS) is the most relevant in aging [24]. Mitochondria have high oxygen consumption rates, and the DNA in these organelles lacks histones for protection. Thus, mitochondrial DNA (mtDNA) is more vulnerable to oxidative damage in the presence of mitROS [25]. Compared to short-lived species, those with longer lifespans tend to generate fewer mitochondrial oxygen radicals, have less unsaturated membrane fatty acids, and experience less oxidative damage to mtDNA [26].

**Figure 2.**
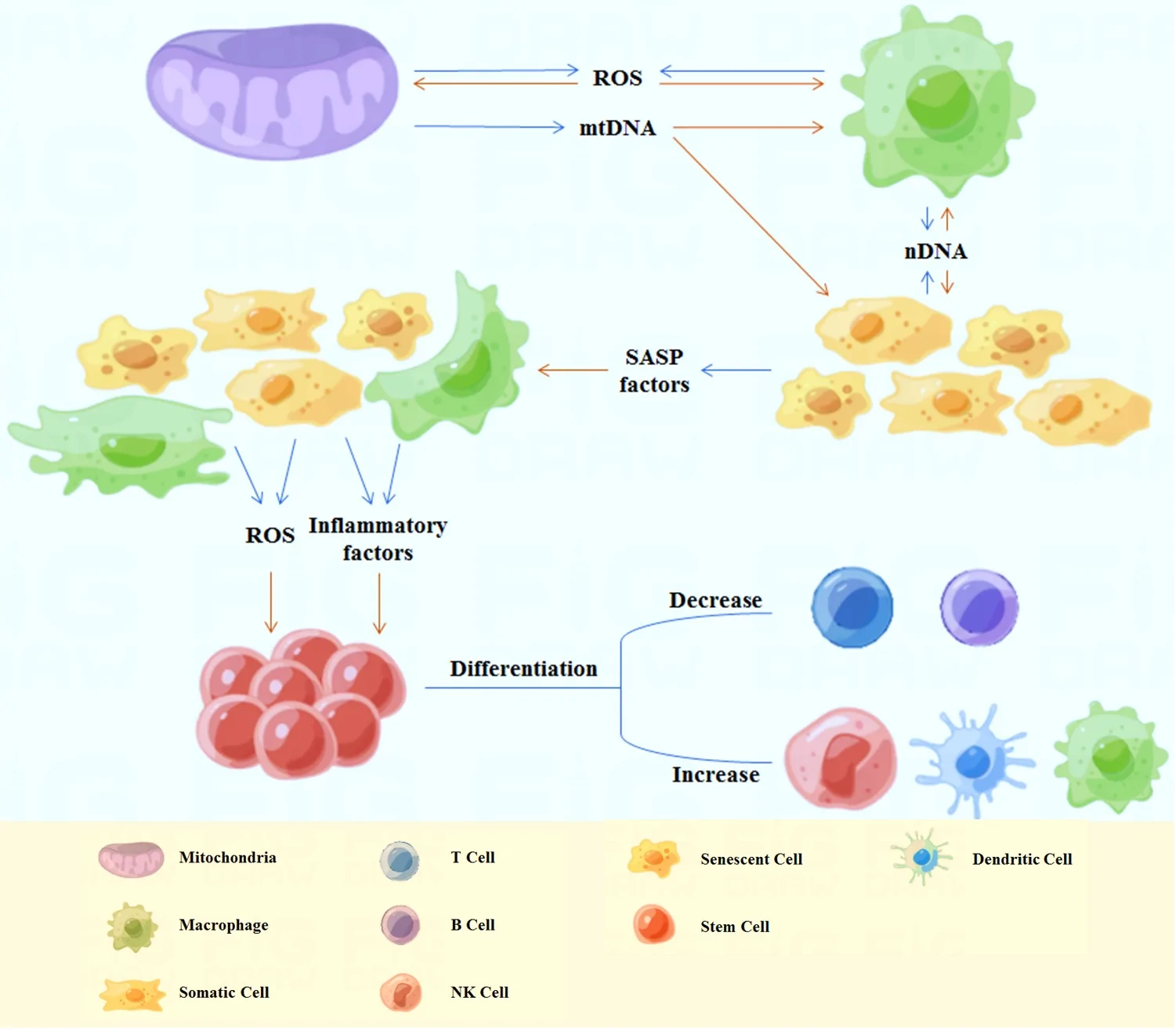
**ROS-inflammation-immune balance**. Mitochondria and macrophages are two main sources of internal and extracellular ROS production. With increasing age, chronic ROS accumulation causes long-term oxidative stress, which exposes cells to a pro-oxidant environment, causing DNA damage, functional loss, and cell senescence. The accumulation of mtDNA and membrane damage caused by ROS disrupts mitochondrial homeostasis and causes the generation of mtDNA fragments, which can travel to other organelles and insert themselves into nDNA, increasing cell damage. Owing to oxidative damage, mtDNA fragments and nDNA fragments can be released into extracellular fluids and blood circulation, impacting cells systemically. Stimulated cells transform into the SASP, through which proinflammatory mediators are released to attract immune cells such as macrophages. Oxi-inflamm-aging occurs when the immune system is activated and is characterized by mononuclear immune cell infiltration into different tissues where these cells produce excessive ROS and inflammatory factors, thereby inducing tissue damage and fibrosis. The exposure of stem cells to excessive ROS and proinflammatory factors leads to impaired differentiation and cell senescence. Impaired and aged HSCs result in the domination of hematopoiesis over lymphopoiesis due to increased numbers of myeloid cells, such as macrophages, dendritic cells and NK cells; moreover, the pool of B and T cells shrinks. These hematopoietic changes indicate increased dependence on the innate immune system rather than acquired immunity.

The accumulation of mtDNA and membrane damage caused by ROS can disrupt mitochondrial homeostasis and subsequently impair cell functions. As proposed by Miquel *et al*., damaged mtDNA is particularly significant in postmitotic cells, where mitochondria cannot be adequately regenerated. ROS produced by the respiratory chain damage the genomes and membranes of differentiated cells that do not undergo frequent mitosis and cannot regenerate organelles [27]. The disrupted mitotic process facilitates the accumulation and amplification of ROS damage, which spreads from the molecular level to the cellular level, marking the onset of the aging process [28]. In addition, oxidative damage to mtDNA can cause the generation of mtDNA fragments, which can migrate to other organelles and integrate themselves into nuclear DNA (nDNA), ultimately increasing cell damage [29]. In support of this theory, mtDNA fragments gradually accumulate in nDNA with age, and this change can be reversed by treatment with rapamycin, which can prolong lifespan [30]. Owing to oxidative damage, mtDNA fragments and even nDNA fragments can be released into extracellular fluids and blood circulation, impacting cells systemically and activating the immune system (Fig. 2) [31, 32].

## 3. Proinflammatory and Anti-inflammatory Balance

Oxidative stress can activate the immune system and stimulate the expression of proinflammatory factors, and immune reactions can produce ROS as a defensive agent. When an imbalance between oxidation and antioxidation occurs, the inflammatory response can be strongly affected by and amplify the damage caused by ROS.

## 3.1. Pro- and Anti-inflammatory Factors

Under normal conditions, the body keeps a state of low entropy through self-organizing systems, and the degree of self-organization depends on the system's ability to exchange substances, energy, and information with the surrounding environment, namely, metabolism. The greater the body's self-organizing capacity, the more effectively it can maintain a state of low entropy, which means maintaining a healthy state and vitality. The metabolic and self-organizing systems are the most vulnerable to diseases, especially infectious and chronic diseases. Chronic non-communicable diseases are responsible for 85% of all deaths in China, and inflammatory aging caused by these diseases increases entropy in the body. Normally, inflammation is a protective response to harmful stimuli and is one of the mechanisms by which damaged tissues are healed. Proinflammatory and anti-inflammatory factors maintain a balance of inflammation in the body. However, excessive inflammation, particularly in the form of cytokine storms, may trigger life-threatening systemic inflammatory syndrome. Similarly, chronic inflammation, which is also known as low-grade or persistent inflammation, is highly harmful because proinflammatory factors can damage human cells and tissues, contributing to a range of chronic diseases, such as heart disease, obesity, type 2 diabetes, and metabolic syndrome.

When activated, immune cells produce proinflammatory mediators, including interleukin (IL)-1, IL-2, IL-6, IL-12, IL-15, IL-18, IL-22, IL-23, tumor necrosis factor (TNF)-α, and interferon (IFN) [33]. Therefore, inflammation by itself is not a negative phenomenon because it plays a crucial role in preserving life [34, 35]. However, it is crucial to regulate this response carefully and ensure it ends shortly after the harmful agent is dealt with, mainly through immune cells activating an anti-inflammatory response. As part of this response, these cells produce anti-inflammatory factors such as IL-1Ra, IL-4, IL-10, and transforming growth factor (TGF)-β1 [33]. Therefore, a balance between proinflammatory compounds, which are needed to address damaging agents and are essential for survival, and anti-inflammatory agents is vital for the immune system to function correctly [36-38].

## 3.2. ROS Trigger an Inflammatory Imbalance

Fragments of mtDNA and nDNA produced by oxidative stress serve as danger signals or damage-associated molecular patterns (DAMPs), which can interact with pattern recognition receptors (PRRs) and trigger nuclear transcription factor kappa B (NF-κB) activation to induce the expression of proinflammatory cytokines [39, 40]. For example, in aging subjects, an increase in ROS induced the excessive expression of TNF-α, IL-6, and IFN in peripheral macrophages [41]. Moreover, mitROS can activate the NACHT, LRR and PYD domain-containing protein 3 (NLRP3) inflammasome, which is responsible for the processing and secretion of proinflammatory cytokines such as IL-1, IL-18, etc [42-45]. Cells that are exposed to excess ROS can transform into the senescence-associated secretory phenotype (SASP) to prevent malignant transformation. In the SASP, proinflammatory mediators are released to recruit immune cells including macrophages, cytotoxic T cells or natural killer (NK) cells [46]. Thus, immune cells are unceasingly stimulated by the effects of chronic oxidative stress, leading to an imbalance between proinflammatory and anti-inflammatory compounds, and the transient beneficial effects of inflammation become chronic and harmful. The loss of genomic integrity and the accumulation of mutations, which increase entropy, are particularly apparent in systems that exhibit chronic inflammation. Inflammation-related processes contribute to increased cellular chaos, immune dysregulation, and loss of homeostasis, all of which further exacerbate the entropic decline of the organism [10].

## 3.3 Development of Oxi-inflamm-aging

Oxidation and inflammation occur during activation of the immune system, marked by the infiltration of mononuclear immune cells into tissues, in which these cells produce excessive ROS and proinflammatory factors, thereby inducing tissue impairment and fibrosis. Therefore, a continuous active oxidant response by immune cells can cause ROS overproduction and subsequent cellular damage, which can attract other inflammatory cells, resulting in more proinflammatory and oxidant production and increasing cellular damage [47, 48]. Oxidation and inflammation act as both the results of and contributors to increased entropy in aging. On the basis of the close connection between inflammation and oxidation, the effects of these two processes on aging are termed oxidative inflammatory aging (oxi-inflamm-aging), which provides a more complete and integrative view of the aging process. Immune cells require the generation of oxidant and inflammatory compounds to perform their defensive tasks; however, they may be responsible for the excessive generation of ROS and proinflammatory factors when overactivated [49]. Oxidative stress and inflammatory stress collectively contribute to the development of chronic low-grade inflammation, and prolonged proinflammatory activation leads to unregulated collateral tissue damage, causes functional deterioration, and results in the loss of tissue homeostasis, leading to progressive organ dysfunction [50, 51]. This chronic low-grade inflammation accelerates physiological aging, increasing the body's overall entropy and contributing to the onset of age-related diseases.

## 3.4. Consequences of Oxi-inflamm-aging

Long-term oxidative-inflammatory stress can harm different types of cells, such as somatic, immune, and stem cells. When they are exposed to excess ROS and inflammatory cytokines, cell cycle checkpoint regulators are up-regulated, halting cell replication progress. As a result, these cells accumulate damage but do not undergo apoptosis. Instead, they upregulate prosurvival pathways called senescent cell antiapoptotic pathways (SCAPs) and downregulate the expression of key apoptotic mediators, leading to the cumulation of senescent cells [52-55]. Moreover, these cells transform into the SASP and activate immune cells, amplifying damage. In particular, the increased number of senescent cells and their secretory effects can cause various potentially negative downstream consequences, such as persistent inflammation, fibrosis, and immune system dysregulation. Aging can be seen as the interplay between increasing entropy and the loss of biological information. This loss of information is not only related to DNA mutations but also to epigenetic changes, which can drive cellular senescence. Recent studies propose that loss of epigenetic information may be a primary cause of aging. These changes manifest in phenomena such as the relocalization of chromatin-modifying proteins during DNA repair, which accelerates cellular aging [56]. The persistence of damage, and the limited capacity for repair over time, aligns with entropy’s tendency to increase disorder in closed systems.

In immune cells, this imbalance results in a broader range of damage across the body. Oxidative inflammaging leads to enhanced adhesion of neutrophils and leukocytes to the endothelium, hindering their migration to infection sites and reduces their chemotactic ability [21, 57]. The dysfunction of these immune cells is believed to result from elevated oxidative stress in both the cells and the endothelium. The production of integrins and cadherins is stimulated by this stress, which improves their binding to the endothelium and curtails their migration [58], resulting in an increase in infection. As individuals age, another impaired function is the phagocytic capacity of neutrophils and macrophages [21, 57]. The decrease in phagocytic capacity leads to the persistence of oxi-inflammatory neutrophils at sites of damage, which can conduce to a failure to resolve inflammation, ultimately resulting in tissue damage and even mortality [59, 60]. Moreover, tissue-resident macrophages play a crucial role in removing other senescent cells [61, 62]. Thus, on the one hand, the decreased phagocytic ability of these cells can increase susceptibility to infectious agents, but on the other hand, it can cause tissue damage and the accumulation of apoptotic and senescent cells in body, leading to the consistent activation of immune cells and increasing oxidation and inflammation[63]. Given the role of the immune system in homeostasis, these changes and reorganizations in immune cells with age are amplified. Immunosenescence is characterized by poor responses to vaccination, a lower capacity to mediate anticancer responses, increased oxi-inflammation, the cumulation of senescent cells, autoimmunity, tissue damage, and the loss of control over infections [4, 64]. An age-related decline in immune cell function contributes to damage by exacerbating inflammation, promoting tumorigenesis, and causing tissue dysfunction [65, 66].

## 4. Innate and Adaptive Immune Balance

Oxidative inflammatory stress can affect cells locally and systemically via paracrine signaling or fluid circulation and can cause cell senescence and inflammaging. In addition to causing the deterioration of somatic cells and immune cells, oxidative inflammatory stress can also affect stem cells. When stem cells are exposed to excessive ROS and proinflammatory factors, numerous systemic effects occur.

The immune system defends the body by monitoring and resisting foreign pathogens, maintaining immune balance, and protecting cells from infection. Immune entropy was previously used to monitor health status. Entropy analysis is used to assess immune-related genetic parameters linked to immune-related diseases and is a useful indicator of the humoral immune response [5]. Reducing immune escape during viral infection can significantly slow this process.

## 4.1. Innate and Adaptive Immunity

Through a highly structured process of progressive lineage commitment, hematopoietic stem cells (HSCs) and hematopoietic progenitor cells (HPCs) continuously produce hematopoietic and immunological cells. Under normal conditions, While HPCs actively multiply and support daily hematopoiesis, HSCs are primarily quiescent under normal conditions. When confronted with hematopoietic challenges, such as life-threatening blood loss, infection, and inflammation, the proliferation of HSCs is activated [67]. These stem cells and progenitor cells can differentiate into two types of immune cells, forming the innate (natural or nonspecific) and adaptive (acquired or specific) immune systems. Innate immunity occurs at birth and provides the first barrier against pathogens. If pathogens succeed in passing the epithelial and mucosal barriers, innate immune cells, including monocytes, macrophages, neutrophils, and dendritic cells, promptly eliminate them to control the infection. Moreover, NK cells play a role in managing infections and tumor resistance [68]. Adaptive immunity offers a complex immune reaction when the body's initial defenses are unable to swiftly remove the infection. Antigen-recognition receptors are present on the surface of lymphocytes, including T cells and B cells. These antigens could be free in the extracellular fluid or delivered by antigen-presenting cells (APCs) [68]. These two immune systems work in an intricate way and form a balance that is responsible for maintaining homeostasis.

## 4.2. Breaking the Innate and Adaptive Immune Balance

Damage, including protein misfolding, lipid peroxidation, and DNA damage, leads to increased disorder within cells, consistent with the principles of entropy. This concept is supported by evidence that aging cells pass on damaged molecules to daughter cells, suggesting that biological aging mechanisms are closely linked to increasing molecular entropy [69]. As oxidative and inflammatory stress accumulate, the innate and adaptive immune balance can be altered [70, 71]. The accumulation of DNA damage in aged HSCs may be caused by increased intracellular ROS and naturally occurring genotoxic metabolites [72-74]. Moreover, these cells show signs of dysregulated intracellular homeostasis, including reduced DNA repair capacity, protein misfolding, abnormal chromatin modifications, enhanced proinflammatory signaling, and upregulation of stress responses [70, 75, 76].

These molecular alterations lead to a shift in hematopoiesis, favoring myeloid lineage production over lymphoid lineage. This is reflected by an increase in myeloid-biased hematopoietic stem cells (HSCs), myeloid progenitors, and myeloid cells, while the pool of B and T cells decreases [67]. With aging, B-cell production undergoes substantial changes. For example, the naïve B-cell pool decreases, whereas the memory B-cell pool increases. Additionally, decreased antibody affinity and poor class switching are linked to a decline in the diversity of the B-cell repertoire. This dysregulation can also lead to the production of autoantibodies, raising the risk of spontaneous autoimmunity [77, 78]. Moreover, thymic involution contributes to the decline in de novo T-cell generation with aging. As naïve T cell niches in peripheral lymphoid tissues become populated, CD8^+^ T cells experience oligoclonal growth and their repertoire is biased toward previously encountered antigens [79]. Although the number of myeloid cells increases during innate immunity, their functionality decreases. NK cells exhibit decreased cytotoxicity and cytokine secretion, neutrophils migrate less in response to stimuli, and macrophages exhibit reduced phagocytic activity and a decreased oxidative burst [80-82]. These hematological alterations lead to a greater reliance on the innate immune system instead of acquired immunity and an increased basal level of inflammation, increasing the risk of myeloid neoplasia or spontaneous anemia [67].

## 4.3. Immunosenescence

Functional deteriorations in the innate and adaptive immune systems are hallmarks of hematopoietic system aging. This immunosenescence increases vulnerability to infections, reduces the effectiveness of vaccinations, and increases the risk of developing autoimmune diseases and hematopoietic malignancies [83, 84]. Moreover, the immune system's capacity to effectively combat cancer cells is compromised [85]. For instance, people over 65 years old account for 80-90% of influenza virus infection deaths, indicating that they are especially vulnerable to the virus [86]. Immunosenescence also affects transplantation, since bone marrow from elder donors has a reduced ability to rebuild the immune system in recipients [87].

Furthermore, the generation of pro- and anti-inflammatory mediators is changed as a result of the age-related loss of immune cells' highly regulated functions. Therefore, oxidative inflammaging may also be caused by immunosenescence [64, 88]. Overproduction of inflammatory mediators by immune cells can lead to a systemic buildup of proinflammatory mediators when defense processes are not strictly controlled. The low-grade chronic inflammation associated with aging and the SASP is triggered by different cell types, with senescent macrophages potentially playing a major role in inflammaging [89, 90]. In the same way as pathogen-associated molecular patterns (PAMPs) combat infections or repair damage, injured and dying cells emit endogenous molecules called DAMPs that stimulate the immune system. After DAMPs attach to PRRs, the transcription factor NF-κB and the inflammasome pathway are activated. This triggers the continuous synthesis of proinflammatory and oxidant chemicals, which damages tissue, causes cellular senescence, and releases DAMPs through a feedback loop [64, 88]. Moreover, a decrease in regulatory CD4^+^ T cells can result in some acute responses not being terminated in a timely manner, resulting in continuous oxidation and inflammation [91]. Moreover, dysfunctional macrophages, neutrophils and NK cells cannot clear senescent cells quickly, and the oxidative inflammatory response is continuously activated and amplified. Therefore, immunosenescence, which refers to the aging of the immune system, results from the aging process and contributes to it by generating oxidant and inflammatory substances that harm and induce aging in other tissues.

In summary, we believe that senescence transforms from a temporary and homeostatic process to a chronic and harmful condition as a result of the immune system's degeneration with age [92]. Therefore, extending the healthy lifespan in the aging population would be greatly aided by postponing or reversing the immune system's aging effects.

## 5. Age-related Diseases

The balance among oxidation, inflammation and immunosenescence that we propose in this paper is an essential axis in body homeostasis. Disruption of this balance can occur in a variety of age-related diseases and pathologies and plays a key role in the occurrence and development of diseases. This imbalance reflects a biological system moving away from equilibrium, with entropy increasing as disease progresses.

### 5.1. Cardiovascular Disease

Cardiovascular diseases (CVDs) include heart failure, myocardial infarction, stroke, arrhythmia, peripheral arterial disease, and atrial fibrillation. Oxidative stress is associated with the development of CVD. ROS can cause mitochondrial dysfunction and protein misfolding, leading to endothelial damage; in addition, immune cell activation promotes plaque formation and progression in atherosclerosis, a key factor in CVD [93, 94]. The oxidation of low-density lipoprotein (LDL) cholesterol in the early stages of atherogenesis can activate the endothelium and trigger the immune response [95]. Monocytes and T cells adhere to the artery intima and migrate into it. Following phagocytosis of oxidized LDL, macrophages emit proinflammatory factors and ROS, which further oxidize LDL [96]. An imbalance in oxidants and inflammation promotes and amplifies the formation of atherosclerosis.

Inflammaging and immunosenescence contribute to CVD. With aging, the expression of proinflammatory phenotype (M1) of macrophages increases, which contributes to upregulated arterial plaque formation and apoptosis. Atherosclerotic plaques are rich in macrophage-derived foam cells, creating an inflammatory environment that leads to further cellular infiltration and fibrous deposition. In addition, oxidized LDL enhances IL-1β expression, contributing to elevated inflammation in the area of the atherosclerotic plaque. The protein kinase C, extracellular regulated protein kinase (ERK) 1 and NF-κB signaling pathways are activated during the development of atherosclerotic plaques with aging [97]. Furthermore, it has been shown that senescent macrophages directly lead to the development of atherosclerotic plaques [98]. This balance facilitates the emergence and advancement of cardiovascular diseases in the elderly.

### 5.2. Neurodegenerative Diseases

Aging is thought to be a significant risk factor for neurodegenerative illnesses and cognitive disorder including Parkinson's disease (PD) and Alzheimer's disease (AD) [99]. The accumulation of ROS, oxi-inflammation and senescent immune cells occur during the development of neurodegenerative diseases.

Aging causes a marked rise in oxidative stress within the nervous system, resulting in a diminished regenerative capacity and functional decline in nerve cells [100]. For example, voltage-gated potassium (K^+^) channel subfamily B member 1 (KCNB1) can undergo moderate oxidation in aged individuals. The oxidation of KCNB1 is exacerbated in AD or following trauma, leading to significant neurodegeneration [101].

Inflammaging also occurs in the central nervous system [102]. In intact aged neurons, persistent inflammation was observed, accompanied by elevated macrophage infiltration and CC chemokine ligand 11 (CCL11) and monocyte chemoattractant protein 1 levels. The regeneration potential of aged nerves is diminished when CCL11 levels rise since it interferes with Schwann cell differentiation [100]. In the cerebrospinal fluid (CSF) of elderly people, elevated levels of inflammatory cytokines such as TNF-α, interferon-γ-induced protein 10 (IP-10), and IL-8 have been found. The immune cell profile undergoes a shift from the T helper 1 (Th1) phenotype to the non-Th1 inflammatory phenotype. This age-related inflammatory phenotypic shift is exacerbated in AD and multiple sclerosis (MS) patients [102].

Neuronal and glial damage are accompanied by an inflammatory response. With aging, astrocytes and microglia, which mediate the central nervous system's innate immune response, become hyperactivated [103, 104]. Most macrophage responses are decreased by senescence, but activated microglia exhibit increased expression of proinflammatory cytokines and decreased chemotactic and phagocytic activities. As aging progresses, cellular processes exhibit increased entropy due to cumulative molecular damage, metabolic wear, and reduced efficiency in cellular repair mechanisms. Several neurological diseases are negatively influenced by the senescence of immune cells and microglia [105].

### 5.3. Type 2 Diabetes Mellitus

Oxidative stress is believed to significantly influence both aging and the development of type 2 diabetes mellitus (T2DM). Elevated glucose and lipid levels result in oxidative stress due to increases in ROS, protein glycation, and glucose autooxidation. Research has shown that the urinary excretion rate of F2-isoprostane 8-iso-prostaglandin F2α (8-iso-PGF2α), which serves as a crucial indicator of oxidative stress, is greater (p < 0.001) in patients with T2DM than in controls [106]. In cases of advanced diabetes, there was a reduction in antioxidants like vitamin E and α-lipoic acid, and in SOD, an enzyme that aids in the neutralization of O_2_^-^ radicals [107]. Elevated free fatty acid levels cause β-oxidation and mitochondrial uncoupling, which upsets the body's equilibrium between oxidation and antioxidation.

Oxidative stress results from high concentrations of ROS are associated to cell senescence and gives rise to the production of proinflammatory factors [108]. In T2DM, where β cells are still functioning and undamaged, ROS can induce oxidative stress in β cells, impairing insulin production. Furthermore, SASP genes, such as IL-1α, IL-1β, IL-6, and TNF-α, are constantly activated in DM state [109]. In addition, Hotamisligil *et al*. reported that insulin signal transduction can be disturbed by pro-inflammatory cytokines, leading to insulin resistance. Moreover, inflammatory factors may induce β-cell death in islet. Diabetes causes glucotoxicity and lipotoxicity, which elevate the production of L-1β, IL-6, and IL-8 within the pancreatic islets [110]. These proinflammatory molecules decrease transcription of the insulin gene and the number of macrophages infiltration. Crosstalk between β-cells and M1 macrophages in the inflammatory micro-environment of pancreatic islets directly causes β-cell dysfunction and contributes to T2DM. Moreover, crosstalk between insulin target organs and the gut-brain-pancreas axis amplifies the inflammatory microenvironment systemically and causes peripheral insulin resistance [111].

### 5.4.*Rheumatoid Arthritis*

Rheumatoid arthritis (RA) is a chronic inflammatory disorder that causes swelling, tenderness, and joint pain. RA is regarded as an autoimmune disease due to the role of the inflammatory response in its progression [112]. In this condition, ongoing inflammation may cause damage to tissues and the breakdown of cartilage and bone.

Free radicals are an important cause of RA. In a study by Mateen *et al*., RA patients presented increased rates of ROS formation, lipid peroxidation, DNA damage, and protein oxidation. The authors concluded that oxidative stress may be related to the chronic nature of this disease [113]. Neutrophils, macrophages, and lymphocytes generate ROS through NOX family. The accumulation of ROS-induced oxidative stress can lead to disruptions in the signaling and proliferation of T lymphocytes, potentially contributing to the pathogenesis of various diseases [114].

In RA patients, maladaptive T cells exhibit characteristics of premature aging, including CD28 accumulation, telomere shortening, reduced DNA repair capacity, and heightened cytokine production [115, 116]. Unlike typical senescent cells, these T cells remain highly active, secreting proinflammatory cytokines such as TNF-α and IFN-γ. This excessive activity drives chronic inflammation and contributes to the development of autoimmune disorders [117, 118].

Immunosenescence, in which APCs and B cells may play a large role, is also a significant contributor to RA. Senescent dendritic cells (DCs) exhibit heightened hyperactivity even in the absence of infection. This hyperactivity means that DCs more prone to phagocytosing and presenting self-antigens to acquired immune cells, contributing to autoimmunity in RA [119]. B cells tend to produce more autoantibodies with age, exacerbating autoimmunity [120]. Senescent CD8^+^/CD28^-^ T cells have also been linked to auto-immune disease, suggesting that senescence of adaptive immune cells plays a significant role in the onset and progression of autoimmunity [121, 122]. Besides RA, immune-senescence is also seen to trigger other autoimmune diseases like systemic lupus erythematosus and MS [123, 124].

### 5.5.*Cancer*

The mechanism of cancer is intricate, and an imbalance in inflammation has been observed under pathological conditions. Cancer cells adjust their metabolic processes to fulfill the elevated energy requirements caused by their accelerated growth and proliferation, resulting in greater ROS production than normal cells to support standard subcellular operations [125]. Higher mitROS levels facilitate cancer development via mtDNA mutations and redox signaling pathways [126], forming a vicious cycle that promotes cancer. It has been confirmed through research that ROS facilitate tumor development by activating oncogenic signaling pathways in cells [127]. Mutagenic DNA lesions caused by ROS can lead to mutations that result in cancer [128]. The DNA biomarkers of oxidative stress include 8-oxo-7,8-dihydro-2’-deoxyguanine (8-oxodG) and 8-nitroguanine, which have been observed in inflammatory cancer tissues associated with various infectious and noninfectious agents [111]. 8-OxodG mutations are prevalent in the mutated p53 gene, confirming the link between oxidative stress and cancer [129]. mtDNA mutations result in upregulated ROS generation by disrupting the electron transport chain [130]. ROS influence inflammation by triggering the NLRP3 inflammasome and proinflammatory cytokines [131, 132].

Inflammation contributes to tumorigenesis by facilitating angiogenesis, invasion, and metastasis, as well as enhancing proliferative and survival signaling [133, 134]. Inflammaging increases the cellular production of proinflammatory cytokines such as IL-1, IL-6, and TNF-α, as well as their plasma levels [135]. Both NF-κB and signal transducer and activator of transcription 3 (STAT3), which have been linked to the development of cancer, have been demonstrated to be activated by these cytokines [136]. It has been demonstrated that TNF-α causes cancer by producing ROS and causing DNA damage [134]. Additionally, the majority of IL-6's target genes contribute to the growth of cancer cells by being engaged in cell survival and proliferation [137]. Cells are exposed to increased levels of ROS and chronic inflammation; thus, oxidative inflammatory stress occurs, resulting in an inflammatory microenvironment.

The immune system is related to the regulation of cancer. Neutrophils, a type of immune cell, have both tumor-promoting and tumor-fighting functions, and their numbers in the bloodstream rise as tumors grow, eventually making up about 90% of the total white blood cell count [138]. In breast cancer, pro-inflammatory monocytes have been shown to promote cancer, meanwhile patrolling monocytes have an antitumor effect. An imbalance can be observed in monocyte count, and this imbalance increases significantly in late-stage cancer. Tumor-associated macrophages (TAMs) are also essential for cancer because they bridge the inflammatory microenvironment and malignant phenotype of tumor cells and are closely related to drug resistance and disease progression. M2 TAMs, which are often found in oral squamous cell carcinoma and can induce angiogenesis, participate in the formation of the tumor microenvironment [139]. Transcriptional modifications in inflammatory blood monocytes are related to decreased T-cell migration, antigen cross-presentation, interferon response, and cytokine stimulation. The mechanism by which tumors are induced and develop is very complex, and the contribution of the inflammatory balance needs to be determined.

### 5.6.*Nonalcoholic Fatty Liver Disease*

Many studies have shown increased rates of nonalcoholic fatty liver disease (NAFLD) among elderly individuals [140]. Individuals with NAFLD often exhibit metabolic syndrome, with symptoms such as excessive visceral fat, hypertension, hyperlipidemia, insulin resistance and increased secretion of proinflammatory cytokines [141]. The exact mechanism of age-related steatosis remains partially unknown; chronic low-level inflammation and macrophages are believed to be related to steatosis, inflammation and fibrosis in NAFLD.

These age-associated changes can be explained by the balance among oxidative stress, inflammation and immunosenescence. The process leading to liver steatosis is not completely known but is linked to the aging of liver cells, which decreases mitochondrial metabolism [142], reduces autophagic flux [143], or mediates chronic inflammation [144, 145]. Macrophages reportedly accumulate in the livers of NAFLD patients, where they transform into a proinflammatory phenotype and secrete inflammatory cytokines and chemokines [146]. This oxidative inflammaging causes further damage to tissues. Mouse experiment have shown that Ly6C^+^ monocytes accumulation plays an important role in the progression from steatohepatitis to fibrosis and is mediated by a CCL2-dependent mechanism [147]. The accumulation of inflammatory factors and senescent cells leads to accumulation of toxic free fatty acids in liver. Chronic NAFLD gradually develops with the continuous accumulation of these factors.

## 6. Promising Antiaging Interventions: Strategies for Maintaining a Low-entropy State in the Body

Entropy increases during aging, leading to structural and functional damage in all the systems. This increase in entropy is a common mechanism in human disease and aging. Therefore, we can redefine antiaging interventions by focusing on entropy. Antioxidants, anti-inflammatory agents, and immune modulators do more than restore specific balances; their ultimate goal is to reduce the disorder within biological systems, thereby maintaining a low-entropy state in cells, tissues, and organs. Similarly, reducing chronic inflammation is not only about rebalancing inflammatory factors but also about lowering the cumulative entropy that drives the progression of inflammatory diseases.

As we have discussed, oxidative stress, inflammaging and immunosenescence constitute the ROS-inflammation-immune balance and are associated with age-related diseases and pathologies, resulting in disability and mortality in the elderly population. Therefore, therapeutic drugs targeting these three processes can decrease entropy and organization and extend the healthy aging lifespan (Table 1, Fig. 1).

## 6.1. Drugs Targeting the Oxidation/Antioxidation Balance

In terms of the balance between oxidant and antioxidant compounds, there are two ways to alter aging: reducing oxidant concentrations and increasing antioxidant concentrations. The mammalian target of rapamycin/peroxisome proliferator-activated receptor-γ complex 1α/β (mTOR/PGC-1α/β) axis drives the mitochondrial biogenesis pathway, which is significantly increased in senescent lung epithelial cells [148]. The activation of this pathway is associated with increased mitochondrial biogenesis, which increases the number of mitochondria, oxidative phosphorylation activity, and mitROS production. Thus, pharmacological inhibition of mTOR complex 1 (mTORC1) with rapamycin restores mitochondrial homeostasis, decreases the production of ROS, and reduces cellular senescence in lung epithelial cells [148]. Moreover, metformin can inhibit the mTOR pathway, activate adenosine 5’-monophosphate (AMP)-activated protein kinase (AMPK), and reduce ROS levels [149]. To prevent oxidative stress, these drugs can decrease the formation of ROS.

Some antioxidant compounds can prevent oxidative stress by neutralizing ROS. It has been reported that melatonin, which is synthesized from 1-tryptophan, decreases the progression of senescence due to its antioxidant properties [150]. Other natural antioxidant products, such as astaxanthin [151], quinic acid [152] and epigallocatechin gallate (EGCG) [153], can extend the lifespan of *C. elegans* under experimental conditions, indicating their potential antiaging effects.

**Table 1:** Promising antiaging interventions: Strategies for maintaining a low-entropy state in the body.

Drugs	Mechanism	Targeted Balance		
		OAB	PAB	IAIB
Navitoclax (ABT-263)	ωtargets BCL-2 family members to clear senescent cells ωincludes senescent HSCs and senescent MuSCs		✓	✓
Rapamycin	ωdecreases ROS production by inhibiting mTORC1 ωreduces the expression of p16 and p21 in CD3^+^ peripheral T cells	✓		✓
Epimedium total flavonoids	ωinhibits inflammaging by enhancing SIRT6 expression and repressing the NF-κB signaling pathway		✓	
Icariin				
Metformin	ωreduces ROS levels by inhibiting the mTOR pathway ωclears senescent cells by inducing autophagy	✓	✓	
Melatonin	ωproduces antioxidant properties	✓		
3-bromo-4,5-dihydroxybenzaldehyde (BDB)	ωinhibits the phosphorylation of NF-κB and the production of IL-6		✓	
Canakinumab	ωexerts anti-inflammatory effects by targeting the IL-1β innate immune pathway		✓	
Dasatinib and quercetin	ωclears senescent cells by altering networks, including ephrin dependence receptor signaling, PI3K/Akt, and BCL-2 members ωreduces the viability of senescent bone marrow-derived murine mesenchymal stem cells		✓	✓
CD153 peptide conjugate	ωpromotes senescent T-cell clearance from tissue			✓
Fisetin	ωdisables SCAPs to clear senescent cells with the SASP		✓	
Astaxanthin	ωproduces antioxidant properties	✓		
Quinic acid				
EGCG				
Fibrate drugs	ωclears senescent cells by autophagy induction and subsequent senolysis		✓	
mTORC1 inhibitors				
BET inhibition				
ATM inhibitors	ωclears senescent cells by autophagy induction, senolysis, and lysosomal acidification		✓	
Zinc supplements	ωproduces antioxidant properties ωmodulates inflammaging by counteracting the activation of proinflammatory mediators, NF-κB, MAPK, and activator protein-1	✓	✓	
Vitamin C, D, E				
Carotenoids				
Polyphenols				

OAB: oxidation and antioxidation balance. PAB: pro- and anti-inflammatory balance. IAIB: innate and adaptive immune balance

## 6.2. Anti-inflammaging Drugs and Agents

Because of the balance between pro- and anti-inflammaging agents, antiaging drugs and agents typically inhibit inflammatory signaling pathways and reduce the production of inflammatory factors. Epimedium total flavonoids (EFs) and icariin (Ica) are among the many agents that can treat inflammaging. Icariin, which is a natural flavanol glycoside, increases sirtuin 6 (SIRT6) expression and inhibits the NF-κB signaling pathway [154]. In addition, zinc supplements; vitamins C, D, and E; carotenoids; and polyphenols have been shown to have antioxidant effects and are potent modulators of inflammaging [155]. These factors counteract the activation of proinflammatory mediators, such as NF-κB, mitogen-activated protein kinase (MAPK), and activator protein-1. Similarly, 3-Bromo-4,5-dihydroxybenzaldehyde (BDB), a naturally occurring substance generated by the red algae *Polysiphonia morrowii*, inhibits the phosphorylation of NF-κB. Additionally, BDB prevents the synthesis of IL-6, which makes it a powerful anti-inflammatory drug [156]. Canakinumab is a human monoclonal antibody that can treat RA [157], and its use has led to a decrease in the recurrence of cardiovascular episodes [158]. Canakinumab targets IL-1β innate immune pathway, therefore having an anti-inflammatory effect [159].

Senescent cells are another important source of proinflammatory agents. These cells are pharmacologic targets for mitigating the impacts of basic aging processes because they can be eliminated by apoptosis-inducing senolytic agents, which have demonstrated advantages in preclinical and clinical models of geriatric decline and chronic illnesses [160]. There are well-known agents, including the flavonoid fisetin [161], and the combination of the Src kinase inhibitor dasatinib and the flavonoid quercetin (D+Q) [162], that act by transiently disabling SCAPs, leading to the self-destruction of senescent cells that have a tissue-damaging SASP. Typically, these agents have multiple pharmacologic mechanisms that function synergistically. For example, D+Q exerts broad-spectrum senolytic activity by interfering with several prosurvival networks, including the ephrin-dependent receptor signaling pathway, PI3K/Akt pathway, and B-cell lymphoma (BCL)-2 family. BCL-2 family members (BCL-2, BCL-XL, BCL-W, etc.), which prevent the activation of proapoptotic mitochondrial signaling cascades, cytochrome C release, and downstream caspase activation, are targeted by other senolytic agents [52, 163]. Navitoclax (ABT-263), A1331852, and A1155463 are among the compounds that exhibit in vitro activity against senescent human lung fibroblasts and umbilical vein endothelial cells, as well as other cell types [164]. Autophagy, which can clear senescent cells, is inhibited with age [165], however, senescent cells are ready for cell death after an autophagic trigger. This process is exemplified by the induction of autophagy followed by senolysis through the use of fibrate drugs [166], metformin [167], mTORC1 inhibitors [168], bromodomain and extraterminal domain (BET) inhibitors [169], and lysosomal acidification with ataxia telangiectasia mutated (ATM) inhibitors [170]. Prompt elimination of senescent cells can efficiently avert the worsening of oxidative stress and inflammation.

## 6.3. Senescent Cell Clearance Therapies

The imbalance in immunity and immunosenescence can also be reduced by inhibiting aging in immune cells and stem cells. In obese adipose tissue, senescent T cell accumulation contributes to inflammation through both local and global effects [171]. The removal of senescent T-cells from adipose tissue and related enhancements in metabolic function were facilitated by immunizing these cells with a CD153 peptide combination. The inhibition of mTOR activity with rapamycin analog in old individuals reduced infection rates and enhanced their response to influenza vaccination, which indicated that immunosenescence could be regulated [172, 173]. Rapamycin therapy decreased blood levels of monocyte chemotactic protein (MCP)-1 and TNF, as well as the expression of p16 and p21 in CD3+ peripheral T cells. This suggests that immunological senescence causes systemic aging through both gain- and loss-of-function pathways [174]. Oral administration of navitoclax (ABT-263) to aged mice depleted senescent cells effectively, involving senescent HSCs in the bone marrow and senescent MuSCs [175]. Notably, this decrease alleviated the aging of the hematopoietic system and restored aged HSCs and MuSCs in typically aged mice. In a similar fashion, D+Q notably lowered the viability of senescent mesenchymal stem cells derived from murine bone marrow [52]. Thus, immunosenescence can be alleviated or reversed by these therapies.

## 6.4. Non-pharmacological Therapies

In addition to pharmacological interventions, non-pharmacological therapies such as nutrition and physical exercise play critical roles in promoting healthy aging by maintaining a low-entropy state in the body. For instance, the Mediterranean diet, abundant in fruits, vegetables, whole grains, and healthy fats such as omega-3 fatty acids, has been linked to reduced chronic inflammation and oxidative damage [176, 177]. In animal-based research, caloric restriction has also been shown to reduce oxidative damage and inflammation, improving mitochondrial function and promoting longevity [178, 179]. Caloric restriction has been particularly effective in upregulating autophagy, a cellular activity that gets rid of malfunctioning organelles and proteins, helping maintain cellular order and reducing entropy [180-182]. Physical exercise is one of the most effective non-pharmacological strategies for reducing entropy and promoting healthy aging [183]. Regular physical activity improves mitochondrial function, enhances oxidative capacity, and promotes mitochondrial biogenesis [184, 185]. Exercise stimulates the expression of antioxidant enzymes such as SOD and catalase, which help neutralize ROS, thereby reducing oxidative stress and entropy within cells [186-188]. All these therapies are essential for reducing oxidative stress, improving mitochondrial function, and maintaining cellular homeostasis, thus counteracting the increase in entropy typically associated with aging.

## 7. Conclusions

Increasing evidence suggests that aging is characterized by an imbalance in inflammatory processes driven by oxidative stress, inflammaging, and immunosenescence, collectively resulting in an ROS-inflammation-immune balance. This imbalance disrupts homeostasis, increases entropy, and contributes to age-related diseases. Oxidative stress underpins aging by promoting inflammation and cellular senescence across biological systems. The resulting oxidative inflammaging damages stem cells and accelerates immunosenescence. This cascade of balance disruptions creates a feedback loop of increasing entropy, leading to aging and aging-related diseases. Achieving a balance between oxidative and antioxidative forces, proinflammatory and anti-inflammatory responses, and innate and adaptive immunity may be key to promoting healthy aging.

Given the multiple effects and unique properties of the inflammatory response, we propose that an impaired inflammatory balance is a central feature of normal aging. Notably, centenarians present markers of systemic inflammation but retain satisfactory physical function because of increased anti-inflammatory responses and decreased ROS production to maintain systemic homeostasis [189]. Long-term health benefits likely arise from achieving the right balance in the inflammatory response. Therefore, we place more emphasis on maintaining balance to slow the process of aging instead of simply strengthening or weakening one aspect of the balance.

An increase in entropy is a common mechanism underlying human disease and aging. ROS-inflammation-immune imbalance significantly modifies the physiological context by increasing entropy. Maintaining a low-entropy state is crucial for preventing and reversing various pathological processes. Understanding how these processes interact to shift biological systems from low to high entropy can offer valuable insights into the mechanisms underlying aging and disease and inform strategies for therapeutic interventions aimed at maintaining a low-entropy state in the body.
